# Inhibitory Role of the Small Leucine-Rich Proteoglycan Biglycan in Bladder Cancer

**DOI:** 10.1371/journal.pone.0080084

**Published:** 2013-11-06

**Authors:** Christian Niedworok, Katharina Röck, Inga Kretschmer, Till Freudenberger, Nadine Nagy, Tibor Szarvas, Frank vom Dorp, Henning Reis, Herbert Rübben, Jens W. Fischer

**Affiliations:** 1 Department of Urology, Essen Medical School, University Duisburg-Essen, Essen, Germany; 2 Institut für Pharmakologie und Klinische Pharmakologie, Universitätsklinikum Düsseldorf, Heinrich-Heine-Universität, Düsseldorf, Germany; 3 Department of Pathology, Essen Medical School, University Duisburg-Essen, Essen, Germany; 4 Department of Urology, Medical University Vienna, Vienna General, Hospital, Vienna, Austria; University of Patras, Greece

## Abstract

**Background:**

Urothelial bladder cancer is the ninth most common cancer. Despite surgical and chemotherapeutic treatment the prognosis is still poor once bladder cancer progresses to a muscle-invasive state. Discovery of new diagnostic markers and pathophysiologic effectors might help to contribute to novel diagnostic and therapeutic options. The extracellular matrix microenvironment shaped by the extracellular matrix critically affects tumor cell and stroma cell functions. Therefore, aim of the present study was to assess the possible implication of the small leucine-rich proteoglycan biglycan in progression of human urothelial bladder cancer.

**Methods and Results:**

For this purpose tumor biopsies of 76 bladder cancer patients with different tumor stages (pTa, pT1-T4) were investigated with respect to biglycan expression and correlated with a long-term (10 years) clinical follow-up. Interestingly, higher biglycan mRNA expression was associated with higher tumor stages and muscle invasiveness. *In* *vitro* knock-down of endogenous biglycan in human urothelial carcinoma cells (J82 cells) increased proliferation, whereas addition of recombinant biglycan and overexpression of biglycan inhibited tumor cell proliferation. In line with this growth-inhibitory effect of biglycan, transplantation of J82 cells after knock-down of biglycan resulted in significantly increased growth of subcutaneous xenograft tumors in nude mice *in* *vivo*. Furthermore, treatment with two anti-proliferative, multi-receptor tyrosine kinase inhibitors—sunitinib and sorafenib—strongly upregulated biglycan expression. Collectively, the experimental data suggest that high biglycan expression is associated with reduced tumor cell proliferation. In accordance, Kaplan-Meier analysis revealed higher 10-year survival in patients with high biglycan mRNA expression in tumor biopsies.

**Conclusion:**

In conclusion, the present data suggest that biglycan is an endogenous inhibitor of bladder cancer cell proliferation that is upregulated in response to anti-proliferative tyrosine kinase inhibitors. In addition, high biglycan expression is associated with favorable prognosis.

## Introduction

Urothelial bladder cancer is the ninth most common human malignant cancer with growing incidence and prevalence [1]. Non-muscle invasive bladder cancer is treated by transurethral resection and adjuvant instillation therapy in case of high grade disease or in cases with multiple, frequently recurring tumors. The low grade non-muscle invasive disease can progress to muscle invasive high risk bladder cancer. The treatment of choice for muscle invasive bladder cancer is radical cystectomy [2,3] which provides still only a low (~50%) 5-year survival. The main cause for this devastating survival is frequently occurring metastatic progression in these patients [6,7]. Cisplatin in combination with gemcitabine represents the first-line adjuvant treatment option for patients with locally advanced or metastatic bladder cancer. New chemotherapeutic strategies, involving receptor tyrosine kinase inhibitors, are currently investigated in preclinical models and in clinical studies [4,5]. Thus, despite the surgical and chemotherapeutic treatment options the severity of the disease and the poor prognosis urge for the discovery of new markers for disease progression as well as new insights into the pathophysiology of bladder cancer progression. 

To this end, we aimed to investigate specific changes in the extracellular micromilieu as defined by the extracellular matrix (ECM) that are associated with the progression of bladder cancer and whether a specific biological activity could be attributed to candidate molecules. ECM is known to affect both, tumor and stromal cells with respect to their phenotypic characteristics that are relevant for cancer progression such as invasion, proliferation and apoptosis. Furthermore, the composition of the ECM micromilieu is likely part of the intercellular communication between tumor and stroma cells. Proteoglycans, key components of the ECM, are becoming increasingly relevant for cancer biology because of a plethora of functions that are critical for tumor-stroma interactions [8]. Proteoglycans are composed of a core protein and a glycosaminoglycan chain that is subject to posttranslational modifications. Both core proteins and glycosaminoglycan chains contribute to the function of the proteoglycans within the ECM. Small leucine-rich proteoglycans (SLRP) are a family of extracellular proteoglycans that are characterized by leucine-rich repeats in the core protein. The SLRPs are secreted into the extracellular space, where they interact with other proteins including growth factors, cytokines [9-11] and transmembrane receptors [12]. Biglycan (BGN) is a member of SLRPs that interacts with transmembrane receptors of the purinergic P2X7 type and with toll-like receptor (TLR) 2 and TLR4 [13,14]. The former are involved in the activation of the inflammasome, whereas the latter cause activation of the innate immune system. These actions attribute immune modulating properties to BGN that could be of relevance for both, carcinogenesis and tumor progression as well as for metastasis. Furthermore, BGN has been shown to contribute to collagen network formation and stability, to fibronectin fibrillogenesis, to fibroblast differentiation and is proposed to contribute to angiogenic responses [15-18]. In summary, BGN likely modulates tumor biology by effecting important control mechanisms, such as immune responses, matrix assembly and modulation of growth factor activity. This is likely achieved by both, direct cellular signaling evoked by BGN as well as by shaping the microenvironment through its effects on collagen and fibronectin matrix formation. 

In line with the fact that BGN exhibits both, inhibitory and promoting effects on tumor cells, controversial roles have been described for BGN in various cancers. For example, in gastric and colorectal cancer as well as in pancreatic adenocarcinoma, BGN expression was found to be correlated with poor prognosis [19-21]. On the other hand, overexpression of BGN was observed in human pancreatic cancer and found to inhibit growth of pancreatic cancer cells *in vitro* [22]. Furthermore, BGN down-regulation was associated with HER-2/neu-mediated oncogenic transformation which was strongly associated with the malignant phenotype of these cells [23]. Therefore, the role of BGN in tumor progression likely depends on the tumor type, stage and differentiation. Accordingly, these questions have to be addressed specifically in the various tumor entities. Furthermore, the responsiveness of BGN to therapeutic interventions has not been investigated yet. Aim of this study was to assess for the first time the expression and role of BGN in patients with bladder cancer.

## Materials and Methods

### Tumor samples

Gene expression analysis was performed in frozen tissue samples of 76 patients (19x pTa, 15x pT1, 14x pT2, 14x pT3 and 14x pT4), who underwent surgical treatment due to urothelial bladder cancer in the University Hospital of Essen between 1990 and 2004. All frozen tissue samples were cut and stained with H&E to verify their tumor content. Only specimens containing ≥ 70% tumor cells were subjected for further analysis. In addition, 20 cases of paraffin-embedded bladder carcinomas (4x pTa - pT4) were assessed using immunohistochemistry. All patients provided an informed written consent and the Ethics Committee of the Hospital approved the study protocol. The primary endpoint of this study was cancer-speciﬁc survival (excluding all other causes of death). Cause of death was obtained from death certiﬁcates. Subjects were followed up from baseline (date of surgery) survey until July 2009. Normal bladder tissue was taken from patients suffering from non-malignant disease (pyeloureteral stricture, neurogenic bladder dysfunction) and served as control for gene expression analysis.

### Immunohistochemistry

Immunohistochemistry was performed based on the protocols provided by the manufacturer. For BGN immunostaining, the sections were pretreated with chondroitinase ABC to expose the epitopes of the BGN core protein as described previously [24]. Briefly, the digestion with chrondroitinase ABC was performed by treating the slides with chondroitinase ABC prior to the incubation with the primary antibody at 37°C for 1 hour. Tissue sections were subsequently incubated for 16 hours at 4°C with rabbit anti-BGN-IgG (1:250 kindly provided by Larry Fisher, National Institute of Dental Research, National Institutes of Health, Bethesda, USA). Sections with omission of the primary antibody served as negative controls. Detection was performed using 3,3'-diaminobenzidine (DAB). Images were captured using a Leica^®^ DM2000 system and representative areas of all stained slides were documented. In addition the percentage of Ki67 positive cells as a marker of proliferating cells was determined in xenograft tumors (rabbit anti-Ki67 IgG, 1:25, Novus Biologicals, Ltd., Cambridge, UK). The mean microvessel density (MVD) was determined as described before [25]. Briefly, a minimum of two separate tissue sections (10 µm) of 6 mice each group were stained using primary rabbit polyclonal CD31 antibody (1:50, Abcam®, Cambridge, UK, ) and secondary Alexa Fluor 568 goat anti rabbit antibody (1:200, Invitrogen, Molecular Probes, Eugene, Oregon, USA). Images of the whole section were taken with a Zeiss Observer Z1 microscope with 10x objective. Subsequently, three highly vascularized areas of 0.49 mm^2^ were chosen to count vessels. Any distinct CD31-positive endothelial cell cluster separate from adjacent vessels was defined as microvessel. The resulting values were averaged for each mouse. 

Analysis of staining intensities was performed using ImageJ 1.46 software (National Institutes of Health, Bethesda, Maryland, USA) as described previously [26].

### Reverse transcription quantitative real-time PCR (RT-qPCR)

Total RNA was isolated from flash-frozen tumor samples using RNeasy total RNA kits (Qiagen, Hilden, Germany). RNA concentration and purity were evaluated by photometric measurement at 260/280nm. 1 µg RNA was used for cDNA synthesis using the QuantiTect Reverse Transcription Kit (Qiagen, Hilden, Germany). PCR reactions were performed using the Platinum® SYBR® Green qPCR SuperMix-UDG (Invitrogen, Karlsruhe, Germany) in the 7300 real-time PCR system (Applied Biosystems, Darmstadt, Germany). The GAPDH gene was used to normalize target gene expression. Relative expression levels were calculated using the 2^−ΔΔCq^ method. The following primer sequences were used: human BGN, forward CTCCTCCAGGTGGTCTATCT, reverse GGTTGTTGAAGAGGCTGATG and human GAPDH, forward GTGAAGGTCGGAGTCAACG, reverse TGAGGTCAATGAAGGGGTC.

### Cell culture

J82 cells were cultured in RPMI medium containing 10% fetal bovine serum. Receptor tyrosine kinase inhibitors sorafenib and sunitinib were used as solution in dimethylsulfoxide (DMSO) at a concentration of 10 µM (sorafenib) and 1 µM (sunitinib) and DMSO was used as control. Cells were seeded at a density of 1x10^5^ cells per well in 6-well culture dishes (Sigma-Aldrich^®^, Hamburg, Germany) resulting in 60-70% confluence of the cultures at the next day. Subsequently, cells were rinsed with phosphate-buffered saline (PBS, Gibco^®^) and cultured in serum-free medium for 6 hours. The medium then was removed and medium containing 10% serum and the respective receptor tyrosine kinase inhibitor was added. 12, 24 and 48 hours after stimulation the supernatant was harvested and used for immunoblotting, while the cells were harvested for mRNA analysis as described before [27]. Purified BGN (recombinant human BGN, R&D Systems® GmbH, Wiesbaden, Germany) was added to the culture at 0.01-1 µg/ml (0.26 - 26 nM). DNA synthesis was analyzed using [^3^H]-thymidine incorporation assay as described before [28]. Additional experiments to determine the effect of BGN on tumor cell proliferation were performed after seeding the cells at 10.000 cells/3.9 cm^2^ plusminus recombinant BGN in 2% fetal calf serum as specified above. After 7 days cell number was determined using a FACS and Flow-Count Fluorospheres (Beckman Coulter, Krefeld, Germany) as internal standard. Three independent experiments were performed, each of them with six replicates. 

### Immunoblotting

Cells were cultured and medium supernatants were harvested at day 7 after lentiviral transduction (see below). BGN isolation and detection by Western blot analysis was performed as described before [29] using the LF159 rabbit polyclonal BGN antibody (1:500, kindly provided by Larry Fisher, National Institute of Dental Research, National Institutes of Health, Bethesda, USA) or anti-BGN (1:1000, Santa Cruz, Heidelberg, Germany). β-tubulin was detected using the mouse anti-β-tubulin antibody ab11323 (1:5000, Abcam®, Cambridge, UK). Quantification was achieved using fluorescent secondary antibodies and the Odyssey Infrared Imaging System (LI-COR Biosciences, Lincoln, USA). Chondroitinase ABC (0.4 U/sample, Sigma-Aldrich®, Hamburg, Germany) was used to remove GAG-chains as indicated. Medium derived from human coronary smooth muscle cells (PromoCell, Heidelberg, Germany) was used as a positive control for BGN immunoblots.

### Lentiviral transduction

J82 cells were cultured to 60-70% confluence. To knock-down BGN in human J82 bladder cancer cells, short hairpin RNA (shRNA) sequence targeting BGN (forward: 5’CCGGGAACATGAACTGCATCGAGATCTCGATGCAGTTCATGTTCTTTTTTG-3’) in the pLKO.1 vector was used (Sigma-Aldrich®, Hamburg, Germany). Scrambled shRNA was used for the control group. Lentiviral BGN overexpression was performed as described previously [16]. Production of vector, culture of human embryonic kidney cells (HEK-293T), harvest of recombinant particles and lentiviral transduction of human J82 urothelial cancer cells was performed as described before for lentiviral overexpression and knock-down of matrix genes [16,26]. BGN proteoglycan overexpression was performed using the complete cDNA of biglycan as referenced before [16]. For *in vivo* experiments J82 cells were transduced at a multiplicity of infection of 1:10 for knock-down. Cells were harvested on day 7 after lentiviral transduction and BGN knock-down or overexpression was verified by RT-qPCR before further functional assays. 

### Xenograft model

Male NMRI nu/nu nude mice were used for xenografting of human J82 tumor cells and analysis of xenograft tumor growth. Each group consisted of 6 animals. The cells (1x10^6^ tumor cells suspended in 100 µl PBS) were injected bilaterally into the flanks of each animal. Subsequently, the animals were monitored for 7 weeks. Measurement of tumor size was performed twice weekly. Animals were then sacrificed and tumor tissue, liver and lung were harvested. Frozen sections were prepared from all tumor samples for BGN staining, lung and liver sections were prepared for H&E staining to search for metastasis. The local Animal Facility as well as the LANUV (Landesamt für Natur, Umwelt und Verbraucherschutz, NRW) approved the animal experiments and the protocols for handling and monitoring.

### Statistical analysis

Gene expression data were analyzed either by analysis of variance and the Bonferroni post hoc test or by Student's *t* test as appropriate. Data are presented as means ± S.E.M. Statistical significance was assigned at the level of *p*<0.05. Data concerning clinical results from patient parameters were lacking normal distribution, indicating the use of non-parametric two-tailed Wilcoxon rank sum test (Mann-Whitney) for paired group comparisons. Analysis of patient survival data were performed using standard Kaplan-Meier algorithm (PROC LIFETEST, SAS-PC 9.3, SAS-INSTITUTE, Cary, USA). For optimal separation of groups with respect to relative BGN expression, variable cut points were evaluated and log-rank test p-values were recorded. Optimal separation of two groups corresponding to a minimal p-value was found at a relative BGN expression value of 0.09. For multivariable analysis Cox proportional hazards regression models were used. All statistical analyses were calculated by using the IBM® SPSS® statistical analysis software (version 18.0, Chicago, IL, USA). 

### Ethics statement

The study protocol and the consent procedure was approved by the Ethics Committee of the University of Duisburg-Essen, Germany and each patient provided a written informed consent. The project number was 07-3537 for storing fresh frozen and paraffinized tumor material and the relevant clinical data. 

The mice experiments were performed in accordance with the rules and standards of the LANUV (Landesamt für Natur, Umwelt und Verbraucherschutz, NRW) and the local Animal Facility of the Heinrich-Heine-Universität Düsseldorf. The LANUV approved the experiments and confirmed the compliance of quality standards. The project is identified by the number 87-51.04.2010.A149. 

## Results

### BGN mRNA expression in human bladder cancer

**Figure 1:**
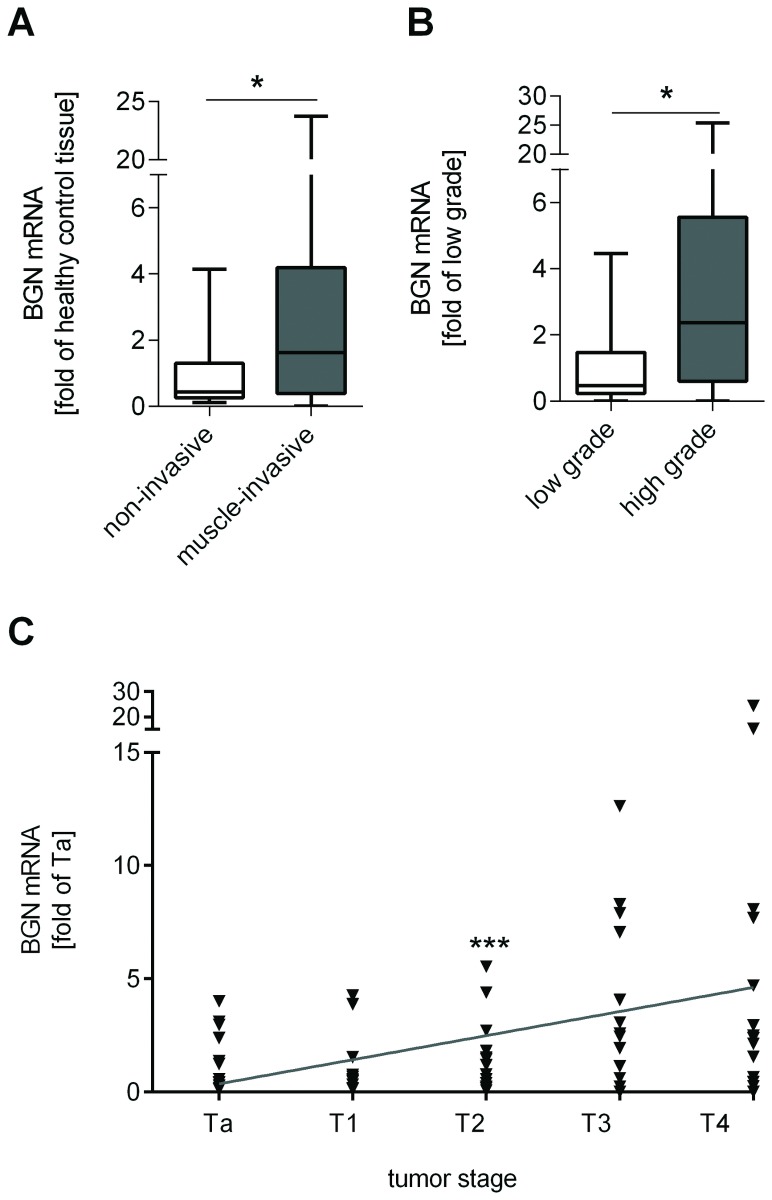
BGN mRNA expression is increased in muscle invasive and in high-grade tumors. BGN mRNA was investigated in 76 patients with bladder cancer by RT-qPCR and compared to non-malignant bladder tissue. A significant elevation of BGN mRNA expression was detected in muscle-invasive compared to non-invasive tumors (**A**) and in high-grade compared to low-grade tumors (**B**). Furthermore, BGN mRNA expression correlated with increasing tumor stage (**C**). n=76 patients, *p<0.05, ***p=0.0003.

First, the samples of 76 patients with different stages of bladder cancer were analyzed with respect to BGN mRNA expression and compared to the expression in healthy bladder tissue. Interestingly, significant differences were detected in mRNA expression levels with respect to tumor invasiveness (Figure **1A**), grading (Figure **1B**) and staging (Figure **1C**), suggesting increased BGN mRNA in progressed stages of bladder cancer. However, despite this positive association with tumor staging multivariate analysis revealed that BGN mRNA expression level (*p*=0.155) was not an independent prognostic factor. In contrast, tumor stage (*p*=0.012) and lymph node involvement (*p*=0.039) were independent prognostic factors for cancer-specific survival as expected (Table **1**). Furthermore, there was no correlation between BGN mRNA expression and time to appearance of metastasis (*p*=0.862), disease progression (*p*=0.624) or disease recurrence (*p*=0.142). BGN was higher in tumor samples of females compared to those of males (4.86-fold versus 1.66-fold of control, respectively, *p*=0.003) and in smokers compared to those of non-smokers (3.40-fold; smokers 2.01-fold, respectively, *p*=0.008, Table **2**).

**Table 1:** Tumor stage and lymph node status are independent prognostic parameters for bladder cancer-specific survival.

	**Cancer-specific survival**		
Variables	HR	95% CI	p
Stage (muscle invasive)	1.488	1.090-2.030	**0.012**
Grade (high-grade)	2.126	0.954-4.740	0.065
Lymphnodes (N+)	2.400	1.045-5.509	**0.039**
BGN (high)	0.946	0.876-1.021	0.155

In a multivariate Cox proportional hazard survival regression analysis in n=76 patients tumor stage and lymph node status were able to predict cancer-specific survival independent from other parameters, *p*-values <0.05 were determined as significant.

**Table 2:** BGN mRNA expression levels are elevated in muscle invasive tumors, high-grade tumors, non-smoking and female gender patients.

		**BGN gene expression**	**P**
		**values**	
	Σ = 76	median (range)	
Age			
≤ 65	35	2.81 (0.06-24.33)	0.847
> 65	**n**	1.95 (0.06-8.28)	
Gender			
Male	59	1.66 (0.06-8.28)	**0.003**
Female	17	4.86 (0.16-24.33)	
Stage			
Ta	19	1.00 (0.13-4.01)	0.986
T1	15	1.06 (0.12-4.26)	0.512
T2	14	1.30 (0.06-5.53)	0.069
T3	14	3.78 (0.23-12.62)	0.909
T4	14	5.21 (0.24-24.33)	
Non-invasive	34	1.03 (0.12-4.26)	**0.004**
Muscle-invasive	42	3.37 (0.06-24.33)	
Grade			
G1	13	0.76 (0.12-4.01)	0.919
G2	27	1.08 (0.13-4.26)	**0.002**
G3	36	3.80 (0.06-24.33)	
Low-grade (G 1-2)	38	0.97 (0.12-4.26)	**0.001**
High-grade (G 3)	37	3.76 (0.06-24.33)	
Lymph node			
N0/Nx	62	2.05 (0.06-24.33)	0.196
N +	14	3.66 (0.23-15.3)	
Primer	43	3.01 (0.12-24.33)	0.883
Recurrent	33	1.44 (0.06-8.08)	
Smoking			
yes	42	2.01 (0.06-24.33)	**0.008**
no	23	3.40 (0.13-15.28)	
unknown	18		

mRNA gene expression levels of n=76 patients with bladder cancer. Data were obtained from flash-frozen tumor samples. Univariate Cox proportional hazard survival regression analysis was used for data analysis, a *p*-value <0.05 was considered as significant.

### BGN protein expression in human bladder carcinomas

**Figure 2:**
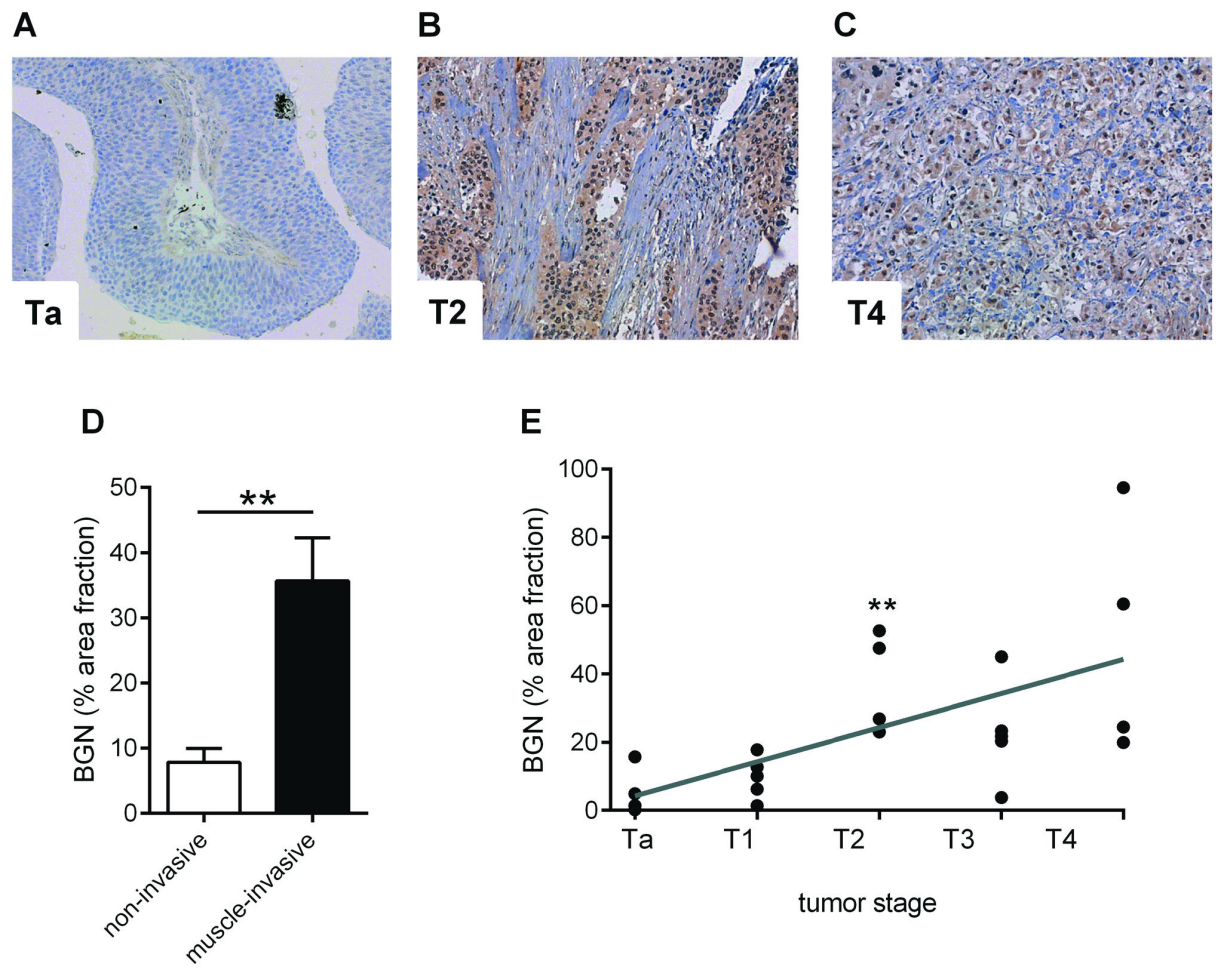
BGN accumulation increases with tumor stage. **A** - **C**, BGN immunostaining was performed on paraffin-embedded tissue sections. (**A**), Ta; (**B**), T2; (**C**), T4. (**D**), quantitative image analysis of BGN in non-invasive tumors (Ta, T1) and muscle-invasive tumor stages (T2-T4). (**E**), correlation of BGN positive area fraction with tumor staging. n=5 patient biopsies of each tumor stage, ***p<0.0001, **p=0.0025.

BGN staining in human tumor samples revealed only faint signals in stromal tissue of non-invasive Ta and T1 tumors (Figure **2A**). BGN staining intensity increased in muscle-invasive tumor stages (Figure **2B and C**). As expected, tumor morphology in superficial stages was similar to normal bladder morphology with a clear differentiation between tumor tissue and stromal tissue. In higher tumor stages (T4), stromal cells and tumor cells were intermixed and both cell types showed association with BGN staining (Figure **2B and C**). However, because BGN is a secreted proteoglycan the cellular source cannot be deduced from immunostaining of tissues. Quantitation of the positive area fraction confirmed the increased staining intensity of BGN in muscle-invasive tumor stages (T2-4) compared to non-muscle invasive Ta and T1 stages (Figure **2D**). In addition, a correlation of the BGN positive area fraction and invasiveness of the tumor was detected (*p*=0.0025, Figure **2E**). 

### Antiproliferative effect of BGN in human bladder cancer cells *in vitro*

**Figure 3:**
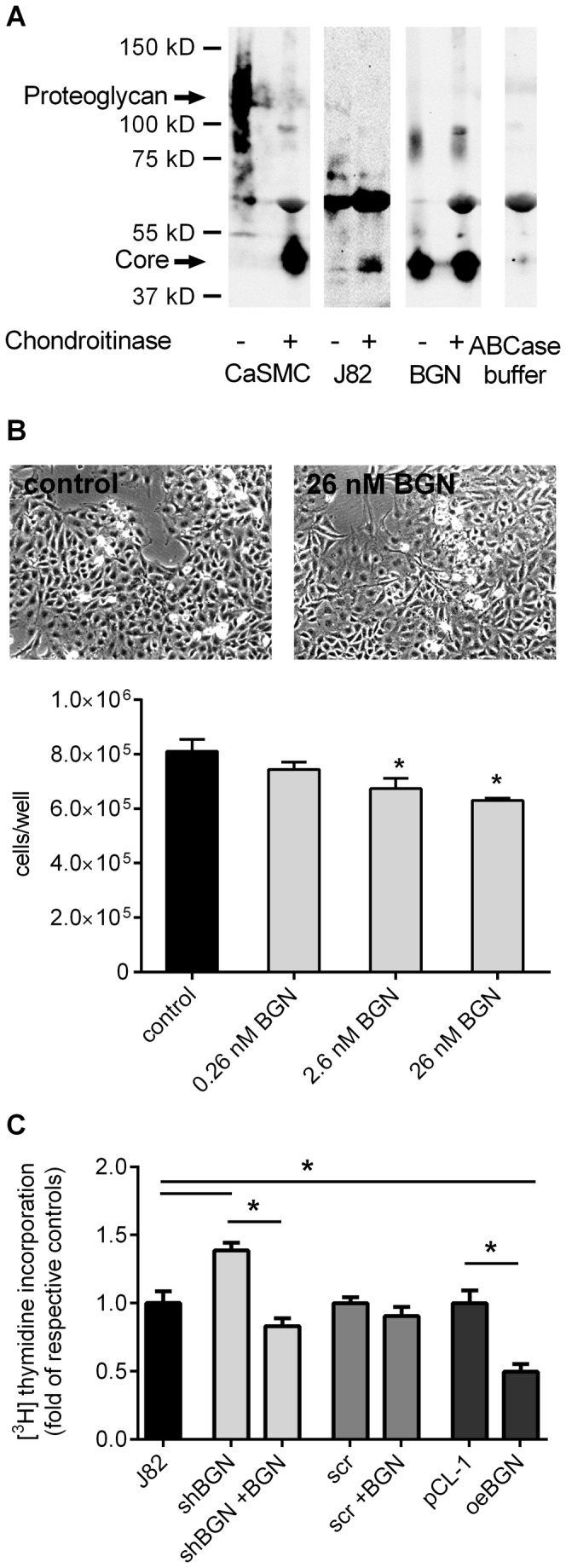
BGN inhibits proliferation in human bladder cancer cells *in* *vitro*. (**A**) Immunoblotting of BGN purified from conditioned media of human J82 bladder carcinoma cells and of recombinant BGN plusminus chondroitinase ABC digestion. As control conditioned medium from human coronary vascular smooth muscle cells (CaSMC) was used because these cells are known to secrete large amounts of BGN proteoglycan (left two lanes). Chondroitinase ABC was applied as additional control (right lane) (**B**) J82 cells were incubated in low serum (2%) with increasing amounts of recombinant BGN and cell number was determined by FACS analysis after 7 days; n=3. Representative light microscopic images are shown. (**C**) Human J82 cells were either lentivirally transduced to knock-down BGN expression or to overexpress human BGN. The respective controls were cells transduced with scrambled shRNA (scr) or empty vector control (pCL-1). In addition, recombinant BGN was used. Nine days after transduction, proliferation was determined as evidenced by DNA synthesis. The BGN knock-down had a proliferative effect that was reversed by addition of recombinant BGN (2.6 nM = 100 ng/ml). Overexpression of BGN resulted in reduced DNA synthesis; n=5, *p<0.05.

The results from human bladder cancer samples suggested that BGN expression associated with tumor progression. To gain mechanistic insights, potential effects of BGN on tumor cell phenotype were investigated *in vitro* as detailed below. First, an immunoblot of secreted BGN was performed from conditioned media of the human bladder cancer cell line, J82 (Figure **3A**). As detected in the sample of J82 cells without chondroitinase ABC digestion BGN proteoglycan was released in relatively small amounts into the medium. In addition, BGN core protein was detected in these samples as well. As a control for cells that secrete large amounts of BGN proteoglycan, the immunoblot of conditioned medium derived from human coronary vascular smooth muscle cells was included (two lanes on the left). Immunoblotting revealed further that recombinant BGN used in the subsequent experiments consisted mainly of core protein because bands were not shifted by chondroitinase ABC (Figure **3A**) and run at the typical size for the core protein. Next, recombinant biglycan core protein was used in a proliferation assay (Figure **3B**). The addition of 0.26 - 26 nM BGN core (0.01-1 µg/ml) caused a dose-dependent inhibition of cell proliferation in J82 cells (Figure **3B**). Finally, to set the basis for an *in vivo* xenograft experiment and to unravel the role of endogenous BGN, lentiviral knock-down of BGN was performed in the human bladder cancer cell line, J82. Importantly, knock-down of BGN caused increased proliferation (shBGN 1.39 ± 0.06-fold of control, *p*<0.05) which was reversed by addition (2.6 nM) of exogenous BGN (0.83-fold of control ± 0.06) (Figure **3C**). The addition of exogenous BGN to cells transduced with scrambled shRNA caused a trend to decreased proliferation. Next, proliferation was determined after overexpression of human BGN. Complimentary to the pro-proliferative effect after BGN knock-down, BGN overexpression resulted in significant reduction of DNA synthesis as compared to the empty vector control (oeBGN, 0.50 ± 0.06-fold of control; pCL-1, 1.0 ± 0.09-fold of control, Figure **3C**).

### Knock-down of BGN accelerates J82 xenograft growth in nude mice

**Figure 4:**
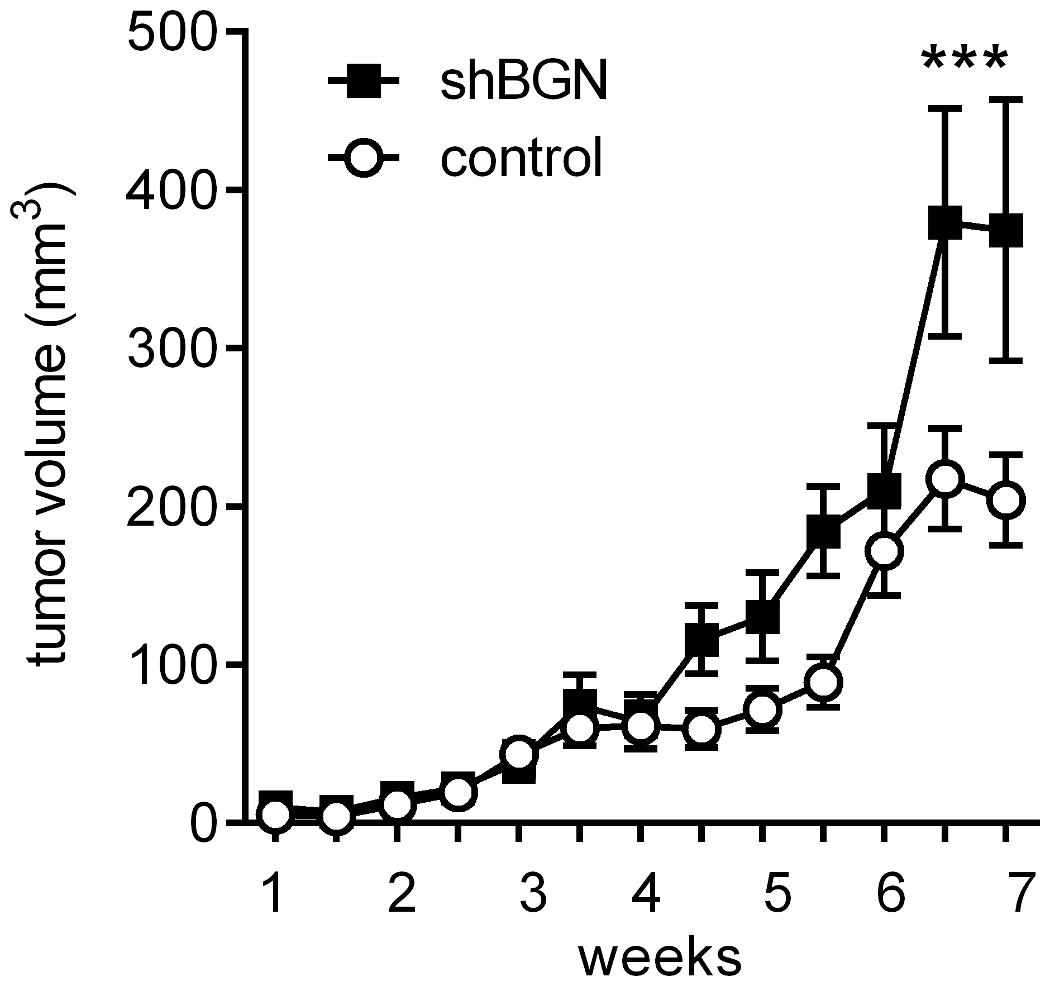
Accelerated growth of xenograft tumors in response to BGN knock-down. After knock-down of BGN in human J82 cells *in* *vitro* the transduced cells were injected bilaterally into the flanks of nude mice and tumor growth was monitored over a period of 7 weeks. As a result the tumor volume was significantly higher after application of J82 cells transduced with shRNA targeting BGN compared to scrambled control (375 mm^2^ vs. 204 mm^2^); n=6, ***p<0.001.

**Figure 5:**
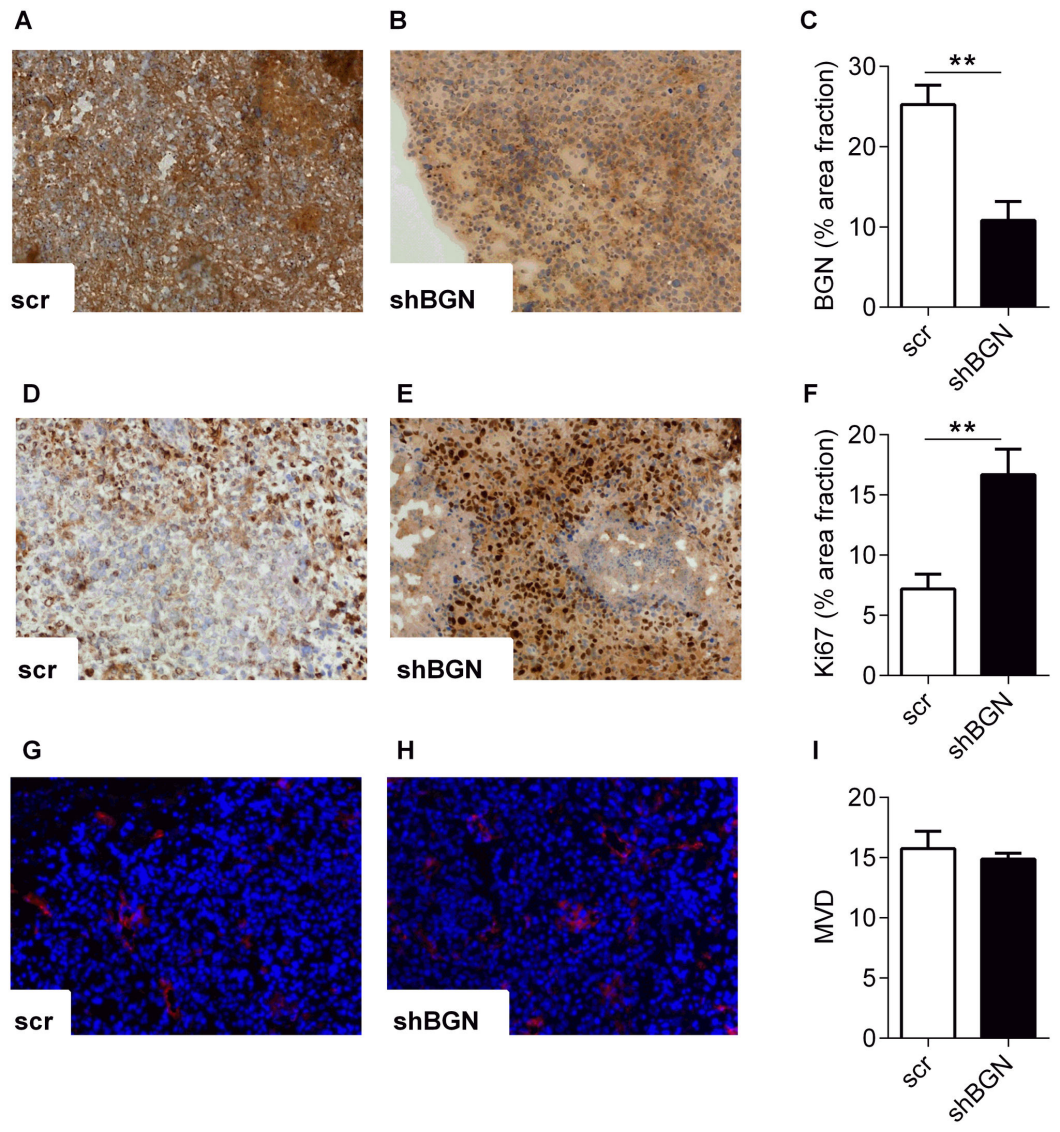
Increased proliferation of tumor cells in response to BGN knock-down in vivo. The xenograft tumors were harvested after 7 weeks and processed for immunostaining and morphological analysis. BGN immunostaining and image analysis revealed decreased BGN expression compared to scrambled control (**A**, **B**). The area fraction of BGN positive staining was 25.3% in controls versus 10.8% in xenograft tumors derived from shBGN cells, n=6, **p=0.0087 (**C**). Next, the proliferative index was assessed using the Ki67 antigen. Compared to control (**D**) the percentage of the area fraction of Ki67 positive cells was significantly elevated in tumors composed of shBGN transduced J82 cells (**E, F**; 7.2% vs. 16.7%, n=6, **p=0.005).(**G**-**I**) The mean microvessel density (MVD) as determined by CD31 immunostaining was not affected in the xenograft tumors, n=6.

J82 cells transduced with shRNA targeting BGN and scrambled shRNA were xenografted into nu/nu nude mice to investigate the effect of BGN down-regulation also *in vivo*. Measurement of xenograft tumor volume revealed significant elevation of tumor growth in the BGN knock-down group compared to the control group treated with scrambled shRNA 49 days after injection of the cells (day 49, shBGN 374.7mm^2^ ± 82.8 mm^2^, scrambled shRNA 204.2 mm^2^ ± 28.5 mm^2^, n=6, *p*<0.001, Figure **4**). The tumors were of firm appearance with no signs of necrosis and only one of the tumors in the shBGN group was infiltrating surrounding tissue (in this case the lower ribs). None of the tumors in the control group was infiltrating the surrounding tissue. There was no visible metastastic growth of tumor cells in the lung and liver as judged by histology. Next immunohistochemical analysis showed that BGN staining still was reduced in the BGN knock-down group (BGN positive area fraction in shBGN group 10.82 ± 2.3% and 25.25 ± 2.4% in scrambled shRNA control group) after 7 weeks (Figure **5 A-C**). Importantly, in line with the *in vitro* data shown in Figure **3**, proliferation of tumor cells was increased after BGN knock-down as evidenced by Ki67 staining compared to control (Ki67 positive area fraction in shBGN group 16.68 ± 2.1% and 7.18 ± 1.2% in scrambled shRNA control group, *p*=0.005, Figure **5D-F**). Tumor vascularization was not affected as evidenced by CD31 staining (Figure 5G-I).

### Multi-receptor tyrosine kinase inhibitors induce BGN

**Figure 6:**
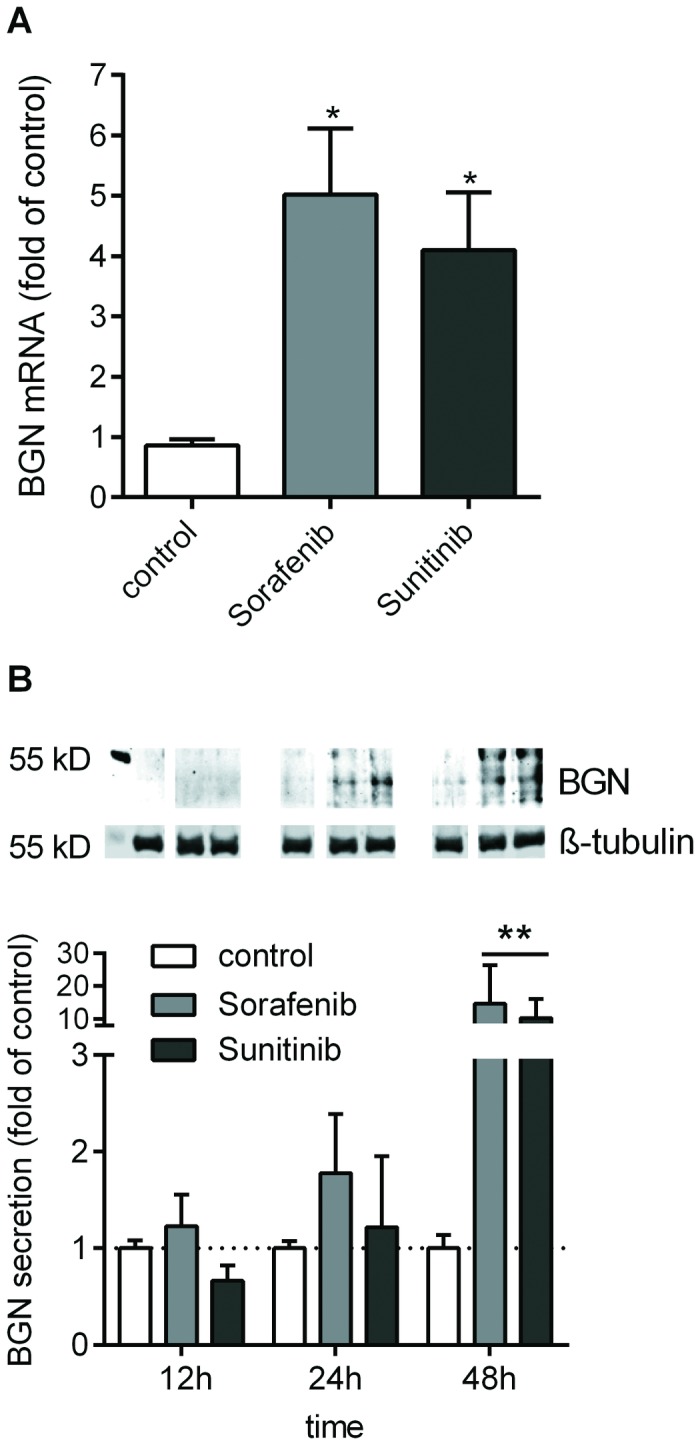
BGN expression is up-regulated after treatment of J82 cells with the multi kinase inhibitors sorafenib and sunitinib. J82 were treated (24 h) with tyrosine kinase inhibitors (sorafenib, 10 µM and sunitinib, 1 µM). (**A**) BGN mRNA expression as determined by RT-qPCR. (**B**), BGN secretion into the cell culture supernatant was determined by immunoblotting after chondroitinase ABC treatment and normalized to the β-tubulin signal of the cell layer; n=4, **p<0.01.

The *in vivo* and *in vitro* data described above strongly suggested that BGN is a growth inhibitor in human bladder cancer cells. Up to date no therapeutic modulators of BGN expression in cancer have been described. Therefore, the effect of small molecule receptor tyrosine kinase inhibitors on BGN expression was investigated. For this purpose the multi-receptor tyrosine kinase inhibitors, sorafenib and sunitinib, were used at concentrations that are known to inhibit proliferation of cancer cells including bladder cancer (J82) cells [30-32]. Cell morphology was not affected within 24 hours of treatment with sorafenib and sunitinib. Of note, treatment with sorafenib (10 µM) and sunitinib (1 µM) resulted in strong up-regulation of BGN mRNA expression after 24 hours (Figure **6A**). In line with the mRNA induction, Western blot analysis of secreted BGN revealed also strongly elevated secretion of BGN 48 hours after treatment with sorafenib and sunitinib (Figure **6B**). These results strongly suggest that treatment with the two multi-kinase inhibitors increased BGN expression and secretion into the medium at concentrations that are anti-proliferative.

### Survival analysis

**Figure 7:**
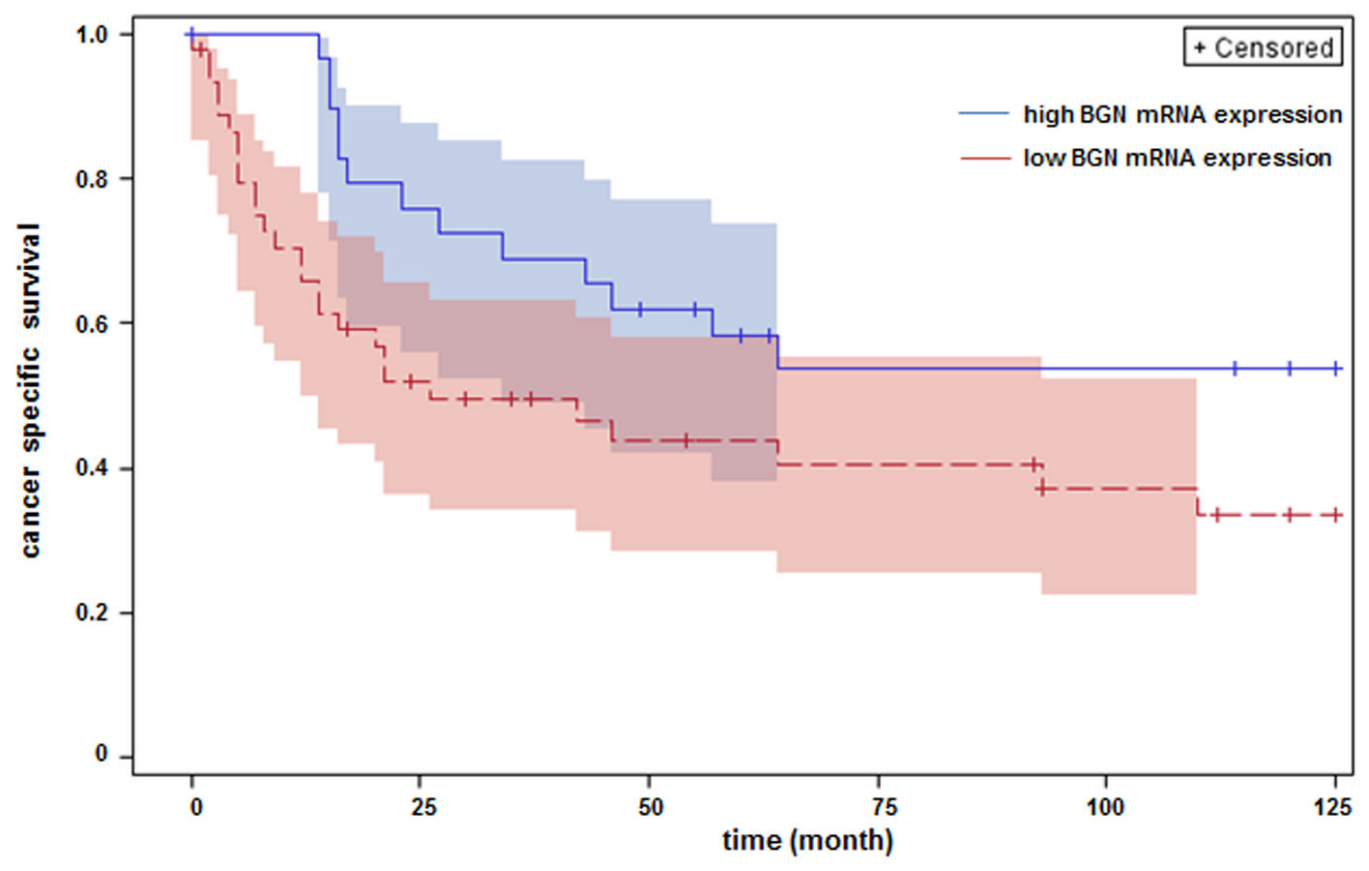
Cancer-specific survival of patients with bladder cancer was discriminated by the extent of BGN mRNA. The 76 bladder cancer patients (see Figure 1) were divided into two groups (BGN high and BGN low) and subjected to Kaplan-Meier survival analysis. For optimal separation of the groups with respect to relative BGN-expression variable cut points were evaluated and log rank test p-values were recorded. Optimal separation of two groups corresponding to the lowest p-value (*p*=0.044) was found at a relative BGN-expression value of 0.09.

The results presented above suggested that BGN is a growth inhibitor of human bladder carcinoma cells although it was found here to be increased in advanced stages of human bladder cancer (Figure **1**). Therefore, it was hypothesized that BGN up-regulation in human bladder cancer tissue is part of an endogenous control mechanism to limit tumor growth. It was tested in a Kaplan-Meier analysis whether patients with high BGN mRNA expression had a better prognosis. For optimal separation of groups with respect to relative BGN-gene expression, variable cut points were evaluated and log-rank test p‑values were recorded. Separation of two groups (BGN high, n = 30; BGN low; n = 45) at relative BGN expression value of 0.09 resulted in a significant p-value of 0.044 (Figure **7**). Deduced from this analysis it appears that high BGN mRNA expression is associated with increased survival of bladder cancer patients. 

## Discussion

Urothelial bladder cancer is one of the most common human cancers. With regard to tumor biology and clinical behavior, urothelial cancers can be divided into two main types: superficial carcinoma and carcinoma invading the muscle layers of the bladder. Due to large differences in clinical prognosis the therapeutic approaches are different in these two types of bladder cancer. Patients with superficial bladder cancer are treated by transurethral resection and in selected high-risk cases additional adjuvant instillation therapy. In contrast, muscle-invasive cancers are treated by radical cystectomy, lymph node extirpation and urinary diversion. For small, unifocal, superficial, low-grade bladder cancers, the estimated 5-year risk of progression to a muscle-invasive tumor is only 0.8%. Recently, several genes have been identified that are associated with bladder cancer progression and poor prognosis, such as excision repair cross-complementing group 1 (ERCC1), matrix metalloproteinase-7 (MMP-7), hyaluronidase 1 (Hyal-1) [33-35]. The latter genes point to an important role of the ECM microenvironment in bladder cancer progression.

The contribution of proteoglycans to the formation and progression of urological cancers has raised increasing interest, because it became clear that proteoglycans are involved in multiple processes, relevant for tumor progression and metastasis, such as responsiveness to growth factors, migration, proliferation, apoptosis, inflammation, angiogenesis and invasive growth [36-38].

The present study reveals for the first time that BGN expression was associated with higher tumor stages (T- and G-staging) in human bladder cancer specimens. Surprisingly, subsequent *in vitro* results in human bladder carcinoma cells revealed that knock-down of BGN accelerated proliferation of cancer cells. Furthermore, addition of recombinant BGN and ectopic overexpression of BGN suppressed bladder cancer cell growth. In accordance, in an *in vivo* xenograft tumor model knock-down of BGN in tumor cells prior to xenografting caused acceleration of tumor growth and tumor cell proliferation. These results strongly suggest that BGN is a suppressor of tumor cell proliferation and tumor growth.

The present analysis revealed that J82 cells expressed BGN both, as proteoglycan and as core protein. For ectopic overexpression the complete cDNA was used that allowed the expression of BGN proteoglycan as well as core protein. Therefore, neither the knock-down of endogenous BGN mRNA nor its overexpression did allow to distinguish between effects of BGN core protein or proteoglycan in J82 cells. The recombinant BGN used in this study, however, consisted mainly of core protein, which strongly suggested that the core protein is sufficient to inhibit J82 proliferation. Since in this study BGN expression was experimentally targeted only in tumor cells, it can be concluded that tumor cell-derived BGN is a growth inhibitor of bladder carcinoma cells. Conclusions about potential contributions of stroma-derived BGN in patients, however, cannot be drawn. 

In line with the hypothesis that BGN is an endogenous inhibitor of bladder cancer progression, Kaplan-Meier analysis revealed improved cancer-specific survival in patients with high BGN mRNA expression. However, this correlation proved not to be independent in the multivariable analysis. Future studies on larger patient cohorts have to further assess the prognostic value of BGN expression in bladder cancer. 

The underlying mechanism responsible for the anti-proliferative effect of BGN in bladder cancer cells *in vitro* and *in vivo* warrants further investigation in the future. It is known that BGN modulates several pathways that are of relevance for bladder cancer progression such as the Wnt/β -catenin pathway, the TGFβ signaling, inflammatory signaling through TLR receptors and the P2X7 receptor. 

It has been shown that BGN activates the Wnt/β-catenin pathway [39]. As Wnt/β-catenin signaling is known to support tumor progression in a variety of tumors including bladder cancer [40-42], the activation of this pathway is unlikely to be responsible for the anti-proliferative effect of BGN in the present study. Based on current data on BGN-mediated modulation of the TGFβ pathway [43] and the stimulatory role of TGFβ1 on bladder cancer cells [44], it can be assumed that effects of BGN on the availability of TGFβ1 may be involved in the anti-proliferative effect of BGN in bladder cancer. 

Another effector of BGN action is the purinergic signaling. Purinergic receptors have been shown to contribute to the growth inhibition of cancer cells [45]. Among them the P2X7 receptor has been attributed anti-proliferative and pro-apoptotic properties. Of note, BGN was recently shown to activate the P2X7 receptor in macrophages and to activate the inflammasome [13]. If the same applies also for bladder cancer cells, it is conceivable that BGN serves as an inhibitor of tumor progression by P2X7-mediated signals. This hypothesis is particularly interesting because BGN is expressed in high amounts by tumor and stromal cells and is relatively stable in contrast to ATP, the principle ligand of all purinergic receptors. 

Furthermore, TLR agonists are being tested clinically for cancer therapy, because they may support immune responses against tumor cells [46]. In addition, TLR2 has recently been suggested to be involved in bladder cancer [47,48]. In this context, it is of interest that soluble BGN serves as a ligand for TLR4 and TLR2 [14], which might explain some of the anti-tumor effects of BGN.

The present results are in line with the literature suggesting the involvement of BGN in tumor biology and progression. However, controversial data have been published regarding the role of BGN in various malignancies. For example, BGN expression was associated with the progression of colorectal cancer [20], gastric cancer [21] and pancreatic adenocarcinoma [19]. In contrast, BGN was shown to induce cell cycle arrest in pancreatic cancer cells by up-regulation of the cyclin-dependent kinase inhibitor p27. Furthermore, in an *in vitro* model of oncogenic transformation of HER-2/neu^+^ cells, down-regulation of BGN enhanced, while overexpression of BGN inhibited HER-2/neu^+^-induced cell proliferation, suggesting BGN as an inhibitor of malignant transformation [23]. The detection of both, association of BGN with malignant properties of tumor cells and poor prognosis and the contrary in other studies including the present investigation, may be due to specific effects of BGN in the different tumor entities and/or tumor stage-dependent effects of BGN. 

Insulin-like growth factor receptor 1 (IGF-R) is upregulated in invasive bladder cancer and activates AKT and MAPK signaling and thereby promotes tumor cell motility [49]. The SLRP decorin, which is closely related to BGN, has recently been studied in bladder cancer [50]. This study provided evidence that decorin is down-regulated and confirmed that IGF-R is up-regulated in high grade bladder cancers. Furthermore, inhibition of IGF-R signaling by decorin was demonstrated. All together, these studies suggested that decorin inhibits expression and activity of IGF-R in bladder cancer and that its loss is permissive for bladder cancer progression to invasive disease. In the present study it is concluded that biglycan is also an inhibitor of bladder cancer progression as suggested from increased tumor progression of mice receiving xenografts after knock-down of BGN and the clinical follow up showing that patients with low BGN mRNA expression have a worse prognosis compared to patients with high BGN mRNA expression in tumor biopsies. In the light of the present findings it is therefore interesting that decorin inhibits motility and that BGN inhibits proliferation of bladder cancer cells. Thus, both SLRPs, decorin and biglycan, appear to be inhibitory but are differentially regulated in bladder cancer. 

The therapeutic exploitation of the potentially inhibitory effects of BGN in bladder cancer is likely impeded by the fact that BGN mimetic strategies are not available. In this regard it is very interesting that *in vitro* treatment of human bladder cancer cells with multi-kinase inhibitors sorafenib and sunitinib resulted in a strong up-regulation of BGN as presented here. Sorafenib is one of the recently evaluated drugs for treatment of bladder cancer patients with advanced disease [32,51,52]. Therefore, it is conceivable that the induction of BGN contributes to the potential anti-tumor effects of sorafenib and sunitinib in bladder cancer. Furthermore, analyzing the tissue samples of the same patients, we have recently identified the receptor of hyaluronan-mediated motility (RHAMM) as an independent predictor of poor survival in human urothelial bladder cancer [53]. It might therefore be considered in the future that the combination of low BGN and high RHAMM mRNA expression is a novel marker pair for the detection of high-risk bladder cancers.

Taken together, this study provides first evidence that BGN (i) is associated with high grade human bladder cancer, (ii) inhibits bladder cancer cell proliferation and progression of experimentally induced xenograft tumors in mice and (iii) is induced by multi-kinase inhibitors. Therefore, tumor-derived BGN-rich extracellular matrix may represent an endogenous growth-limiting mechanism in bladder cancer.
